# Leptin Increases Particle-Induced Osteolysis in Female ***ob/ob*Mice**

**DOI:** 10.1038/s41598-018-33173-9

**Published:** 2018-10-04

**Authors:** Kenneth A. Philbrick, Adam J. Branscum, Carmen P. Wong, Russell T. Turner, Urszula T. Iwaniec

**Affiliations:** 1 Skeletal Biology Laboratory, School of Biological and Population Health Sciences, Oregon State University, Corvallis, OR 97331 USA; 2 Biostatistics Program, School of Biological and Population Health Sciences, Oregon State University, Corvallis, OR 97331 USA; 3 Center for Healthy Aging Research, Oregon State University, Corvallis, OR 97331 USA

## Abstract

Particles generated from wear of prosthesis joint bearing surfaces induce inflammation-mediated periprosthetic bone resorption (osteolysis). Morbidly obese leptin-deficient *ob/ob* mice are resistant to polyethylene particle-induced bone loss, suggesting that leptin, a hormone produced by adipocytes that circulates in concentrations proportional to total body adiposity, increases osteolysis. To confirm that particles induce less osteolysis in leptin-deficient mice after controlling for cold stress (room temperature)-induced bone loss, *ob/ob* mice on a C57BL/6 (B6) background and colony B6 wildtype (WT) mice housed at thermoneutral temperature were randomized to control or particle treatment groups (N = 5/group). Polyethylene particles were implanted over calvaria and mice sacrificed 2 weeks later. Compared to particle-treated WT mice, particle-treated *ob/ob* mice had lower osteolysis score, less infiltration of immune cells, and less woven bone formation. To determine the role of leptin in particle-induced osteolysis, *ob/ob* mice were randomized into one of 4 groups (n = 6–8/group): (1) control, (2) particles, (3) particles + continuous leptin (osmotic pump, 6 μg/d), or (4) particles + intermittent leptin (daily injection, 40 μg/d). Leptin treatment increased particle-induced osteolysis in *ob/ob* mice, providing evidence that the adpiokine may play a role in inflammation-driven bone loss. Additional research is required to determine whether altering leptin levels within the physiological range results in corresponding changes in polyethylene-particle-induced osteolysis.

## Introduction

Joint replacement is highly effective in treating a variety of degenerative joint diseases and restoring function following bone fracture. However, approximately 9% of knee replacements and 15% of hip replacements require surgical revision^1^. Osteolysis induced by wear particles contributes to orthopedic implant loosening and subsequent prosthetic failure^1^. Obesity increases the risk of orthopedic joint failure but the underlying mechanisms remain unclear^2–6^. Excessive weight likely affects the skeleton through increased mechanical loading of joints, but adipocyte-derived factors (adipokines) could have additional actions on bone cells^7^. The adipokine leptin circulates in concentrations proportional to total fat mass^8,9^, and plays a role in energy balance, thermoregulation, regulation of bone growth and turnover, and immune function. Leptin-deficient *ob/ob* mice are resistant to periprosthetic bone loss^10^, suggesting that the adipokine may also play an important role in the etiology of prosthetic joint failure.

*ob/ob* mice are morbidly obese as a result of hyperphagia and reduced energy expenditure. Despite excess weight, these mice exhibit lower total body bone mineral content and bone mineral density^11^ and have shorter^11–13^ and biomechanically weaker^14^ long bones compared to wild type (WT) mice. However, cancellous bone mass is often greater in lumbar vertebra of adult *ob/ob* mice. The skeletal abnormalities in these animals are closely associated with reduced bone growth and bone- and bone-compartment specific changes in bone turnover balance^11,13,15^. Long-duration (up to 30 weeks) leptin treatment using hypothalamic gene therapy restores a WT skeletal phenotype in *ob/ob* mice^13^. Specifically, leptin increases longitudinal bone growth and the overall rate of cancellous bone turnover, and following normalization of bone mass and architecture, returns cancellous bone turnover balance to normal^7,11,13,15^.

Leptin has the potential to affect bone cells via direct and indirect pathways. Leptin is an immune modulator and cytokines produced by immune cells influence bone turnover. Leptin’s importance in inflammation is illustrated by the elevated incidence of infection-related deaths in leptin-deficient children^16^. At the other extreme, hyperleptinemia is associated with increased obesity-related inflammatory response, which may contribute to the etiology of several obesity-associated diseases, including type 2 diabetes, cardiovascular disease, and arthritis^17^. Chronic inflammation increases bone turnover and is also a strong risk factor for pathological bone loss^18,19^. In spite of these associations, the potential role of leptin in mediating bone loss associated with inflammation has received little attention.

We conducted studies to: (1) characterize the skeletal response to polyethylene particle challenge in leptin-deficient *ob/ob* mice after controlling for cold temperature (room temperature) stress and (2) determine if administration of leptin acts to increase particle-induced osteolysis in these mice. The animals were housed at thermoneutral (32 °C) to prevent skeletal changes induced by standard room temperature housing^20^. Particles were surgically implanted over calvaria and leptin administered subcutaneously. Placement of particles on top of the calvarium models the inflammation and osteolysis induced in humans by orthopedic wear particles^21–29^.

## Results

### Experiment 1: Effect of leptin deficiency on particle-induced osteolysis in mice housed at thermoneutral temperature

**Figure 1:**
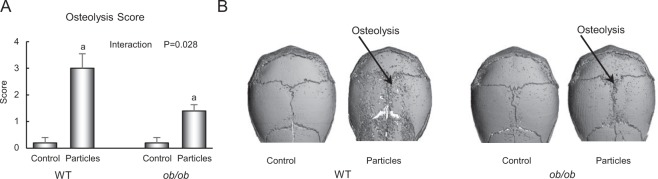
Effects of genotype and polyethylene particle placement over calvaria on calvarial osteolysis in 7-week-old female mice. (**A**) Placement of particles over calvaria resulted in lower osteolysis score in *ob/ob* mice compared to WT mice. (**B**) Representative µCT images of calvaria from WT and *ob/ob* mice. The attenuated osteolysis (less extensive pitting of the calvarial surface) is readily appreciated in the particle-treated *ob/ob* mouse. Data are mean ± SE; n = 5/group. ^a^Different from control within genotype, P < 0.05.

The effect of genotype and polyethylene particle placement over the calvarium on calvarial osteolysis score in WT and *ob/ob* mice is shown in Fig. 1. Placement of particles over the calvarium resulted in osteolysis in both genotypes (Fig. 1A). However, the osteolytic response was greater in WT mice than in *ob/ob* mice. Genotype differences in the magnitude of calvarial osteolysis following particle insertion can be readily appreciated in µCT images from representative WT and *ob/ob* mice (Fig. 1B).

**Figure 2:**
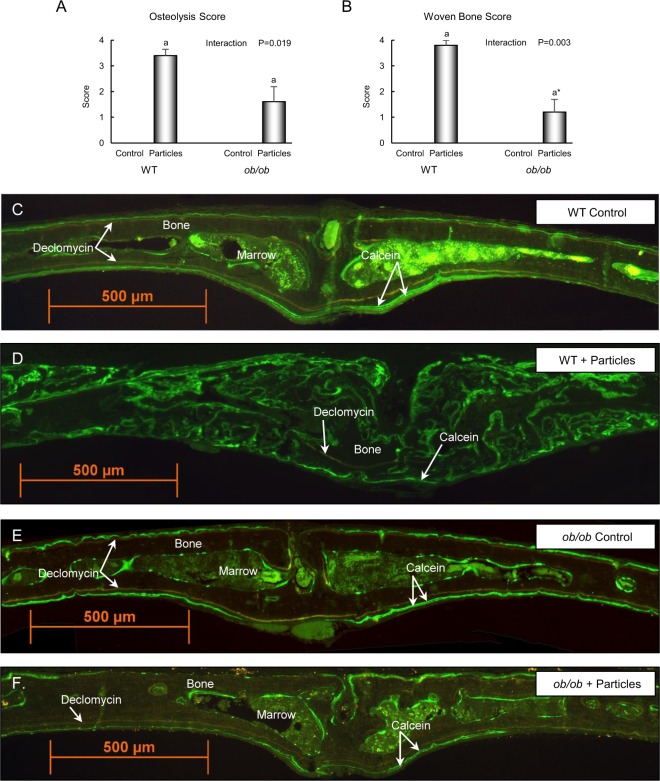
Effects of genotype and polyethylene particle placement over calvaria on calvarial osteolysis and woven bone formation in 7-week-old female mice. Placement of particles over calvaria resulted in osteolysis (**A**) and woven bone formation (**B**) in both genotypes; however, the response was greater in WT mice than in *ob/ob* mice. Photomicrographs of unstained histological sections viewed under ultraviolet light from representative calvaria of a control WT mouse (**C**), a particle-treated WT mouse (**D**), a control *ob/ob* mouse (**E**), and a particle-treated *ob/ob* mouse (**F**) depict the changes induced in response to particle challenge. Evidence of minimal periosteal bone resorption in the WT mouse and *ob/ob* mouse is illustrated by continuous declomycin labeling on both periosteal surfaces. Periosteal bone formation occurring at the time of surgery continued uninterrupted until sacrifice, as illustrated by extensive triple parallel labeling (declomycin, calcein, calcein) in the periosteum. Particles induced extensive bone resorption as illustrated by the absence of the orange declomycin label on both periosteal surfaces in the particle-treated WT mouse. The absence of parallel declomycin and calcein labels provides evidence that bone formation occurring at time of surgery and sacrifice was discontinuous. Particles induced minimal bone resorption in *ob/ob* mice as illustrated by continuous declomycin labeling on both periosteal surfaces. Periosteal bone formation occurring at time of surgery continued uninterrupted until sacrifice, as identified by triple parallel labeling (declomycin, calcein, calcein) on the periosteum. Data are mean ± SE; n = 5/group. ^a^Different from control within genotype, P < 0.05. ^a*^Different from control within genotype, P < 0.1.

**Figure 3:**
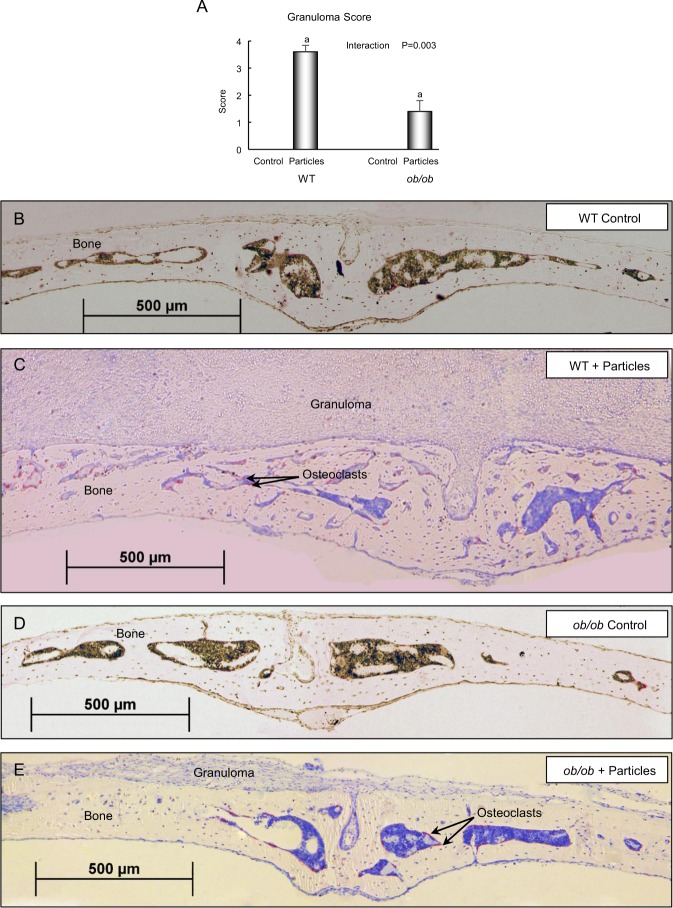
Effects of genotype and polyethylene particle placement over calvaria on calvarial granuloma formation in 7-week-old female mice. Placement of particles over calvaria resulted in granuloma formation (**A**) in both genotypes; however, the response was greater in WT mice than in *ob/ob* mice. Photomicrographs of stained histological sections from representative calvaria of a control WT mouse (**B**), a particle-treated WT mouse (**C**), a control *ob/ob* mouse (**D**), and a particle-treated *ob/ob* mouse (**E**) depict the changes induced in response to particle challenge. Granuloma, characteristic of inflammation, was not present in control WT or control *ob/ob* mice. Particles induced granuloma tissue on top of the periosteal surface in WT mice. Particles also induced extensive bone resorption as illustrated by the presence of osteoclasts (red TRAP-stained cells) in the woven bone. Particles also induced granuloma tissue formation on top of the periosteal surface in *ob/ob* mice, but the response was attenuated compared to WT mice. Data are mean ± SE; n = 5/group. ^a^Different from control within genotype, P < 0.05.

We next evaluated extent of osteolysis, woven bone, and granuloma tissue formation in response to treatment in histological sections of the calvaria (Figs 2 and 3). Placement of particles over calvarium resulted in osteolysis, woven bone formation, and granuloma formation in both genotypes. However, for all three endpoints evaluated, the response was greater in WT mice than in *ob/ob* mice (Figs 2A,B and 3A, respectively).

Photomicrographs of unstained histological sections from representative calvaria of a control WT mouse (Fig. 2C), a particle-treated WT mouse (Fig. 2D), a control *ob/ob* mouse (Fig. 2E), and a particle-treated *ob/ob* mouse (Fig. 2F) illustrate changes induced in response to particle challenge. Periosteal bone formation in WT mice, *ob/ob* mice, and particle-treated *ob/ob* mice continued largely uninterrupted from surgery to sacrifice; this is illustrated by extensive triple parallel fluorochrome labeling (declomycin injected at surgery and calcein injected 4 days prior to sacrifice and 1 day prior to sacrifice). In contrast, particle-treated WT mice exhibited evidence of extensive post-surgical bone resorption (osteolysis) as indicated by much less declomycin-labeled bone. The absence of parallel declomycin and calcein labels indicates that bone formation at time of surgery and prior to sacrifice was discontinuous in these mice. Also, particle-treated WT mice exhibited extensive woven bone. At sacrifice, this bone was undergoing rapid turnover as illustrated by calcein labeling and osteoclast infiltration (Fig. 3C) of the bone. In marked contrast, particle-treated *ob/ob* mice exhibited little woven bone formation.

Photomicrographs of stained histological sections from representative calvaria of a control WT mouse (Fig. 3B), a particle-treated WT mouse (Fig. 3C), a control *ob/ob* mouse (Fig. 3D), and a particle-treated *ob/ob* mouse (Fig. 3E) illustrate the extent of granuloma tissue formation in response to particle challenge. Extensive granuloma superior to the periosteum is clearly evident in the particle-treated WT mouse. Although granuloma is also present in the particle-treated *ob/ob* mouse, the response is attenuated.

### Experiment 2: Effect of leptin treatment on particle-induced osteolysis in *ob*/*ob* mice housed at thermoneutral temperature

**Figure 4:**
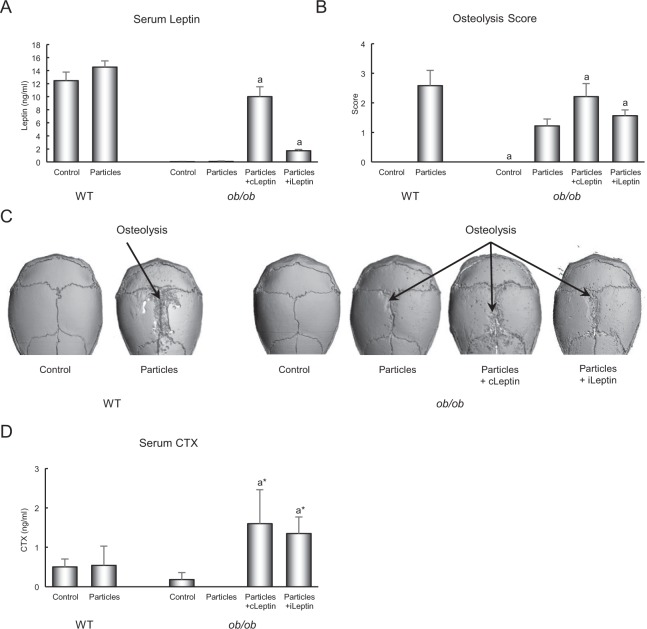
Effects of 2 weeks of leptin administration (continuous, cLeptin; intermittent, iLeptin) on serum leptin (**A**), calvarial osteolysis score (**B**) with representative images in (**C**) and serum CTX (**D**) in 8-week-old female *ob/ob* mice. Control and particle-treated WT mice are shown as a reference. The increase in calvarial osteolysis in *ob/ob* mice in response to leptin is readily apparent in panel C. Data are mean ± SE; n = 6–8/group. ^a^Different from *ob/ob* + particles, P < 0.05; ^a*^Different from *ob/ob* + particles, P < 0.1.

The effects of polyethylene particle placement over calvarium and leptin administration on serum leptin levels and calvarial osteolysis in *ob/ob* mice are shown in Fig. 4. As expected, serum leptin was not detected in the leptin-deficient *ob/ob* mice and administration of leptin, either continuously or as a daily bolus, resulted in measurable levels of the adipokine (Fig. 4A). As in Experiment 1, placement of particles over calvarium resulted in osteolysis in treated mice (Fig. 4B). The osteolytic response was greater in leptin-treated *ob/ob* mice compared to particle only *ob/ob* mice, irrespective of route of leptin administration. Representative µCT images of calvaria from all treatment groups are shown in Fig. 4C; increased osteolysis is clearly evident with leptin treatment in the *ob/ob* mice. The higher osteolysis score in the leptin-treated mice was associated with a tendency (P = 0.082) for higher circulating levels of CTX, a global marker of bone resorption (Fig. 4D).

**Figure 5:**
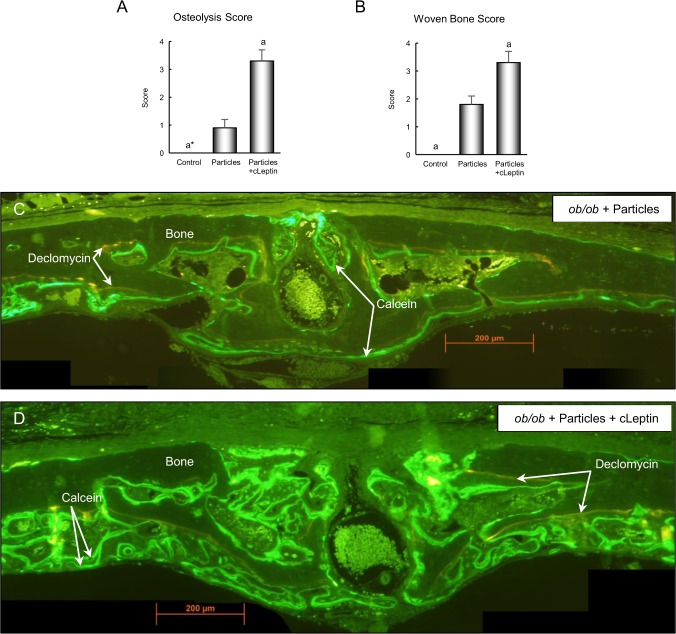
Effects of 2 weeks of continuous leptin (cLeptin) treatment on calvarial osteolysis and woven bone formation in 8-week-old female *ob/ob* mice. Leptin administration resulted in greater osteolysis (**A**) and greater woven bone formation (**B**). Photomicrographs of representative unstained calvarial histological sections, viewed under ultraviolet light, from a particle-treated o*b/ob* mouse (**C**) and a particles + continuous leptin (cLeptin)-treated *ob/ob* mouse (**D**) depict the changes induced by leptin treatment. The increase in particle-induced osteolysis with leptin administration is illustrated by the presence of less periosteal declomycin label, abrupt intersections between declomycin-labeled bone and calcein-labeled woven bone, and the presence of greater woven bone in the particles + cLeptin-treated *ob/ob* mouse. Data are mean ± SE; n = 6–8/group. ^a^Different from *ob/ob* + particles, P < 0.05; ^a*^Different from *ob/ob* + particles, P < 0.1.

**Figure 6:**
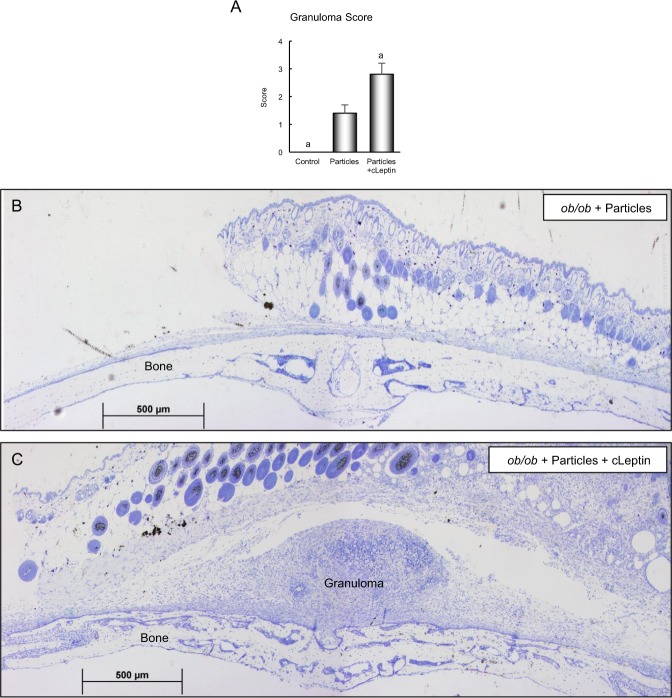
Effects of 2 weeks of continuous leptin (cLeptin) administration on calvarial granuloma tissue in 8-week-old female *ob/ob* mice. Leptin treatment resulted in greater granuloma formation (**A**). Photomicrographs of representative stained calvarial histological sections from a particle-treated o*b/ob* mouse (**B**) and a particles + continuous leptin (cLeptin)-treated *ob/ob* mouse (**C**) depict the changes induced by leptin treatment. Leptin treatment increased particle-induced inflammation as illustrated by the presence of granuloma tissue over the calvarium in the particles + cLeptin-treated *ob/ob* mouse. Data are mean ± SE; n = 6–8/group. ^a^Different from *ob/ob* + particles, P < 0.05.

Osteolysis, woven bone formation, and granuloma formation were subsequently evaluated in histological sections in control *ob/ob* mice, particle-treated *ob/ob* mice, and particle-treated *ob/ob* mice administered continuous leptin (Figs 5 and 6). Placement of particles over the calvarium resulted in osteolysis, woven bone formation, and granuloma formation in both genotypes (Figs 5A,B and 6A, respectively). However, for all endpoints evaluated, the response was greater in the *ob/ob* mice treated with continuous leptin compared to particle-only mice. The response to continuous leptin in particle-treated *ob/ob* mice is readily apparent in Figs 5D and 6C.

Particle treatment in *ob/ob* mice had no significant effect on terminal body weight (Supplemental Figure S1A), lean mass (Supplemental Figure S1C), fat mass (Supplemental Figure S1D), or abdominal white adipose tissue (WAT) weight (Supplemental Figure S1F). However, weight gain over a 10-day period (4 days post-surgery to sacrifice on day 14) was lower (Supplemental Figure S1B) and percent body fat (Supplemental Figure S1E) tended to be lower (P = 0.077) in particle-treated *ob/ob* mice compared to control mice. Leptin treatment in *ob/ob* mice, irrespective of route of administration, resulted in lower fat mass and percent body fat, and higher lean mass and uterine weight (Supplemental Figure S1G) compared to particle-only *ob/ob* mice. In contrast, terminal body weight and abdominal WAT weight were lower in *ob/ob* mice treated with continuous but not intermittent leptin compared to particle only *ob/ob* mice. Continuous leptin administration resulted in weight loss whereas intermittent leptin administration resulted in attenuation of weight gain.

Particle treatment had no significant effect on blood glucose in *ob/ob* mice (Supplemental Figure S1H) but resulted in lower serum corticosterone levels (Supplemental Figure S1I). Blood glucose and serum corticosterone were lower in particle-treated *ob/ob* mice administered leptin, irrespective of route of administration, compared to particle-only *ob/ob* mice.

## Discussion

*ob/ob* mice housed at either room temperature^10^ or at thermoneutral temperature (present study) were resistant to polyethylene particle-induced osteolysis. At the cellular level, polyethylene particles placed over calvaria of WT mice induced granuloma tissue infiltration, extensive bone resorption, and disorganized bone formation. In contrast, bone resorption and granuloma were less apparent in particle-treated leptin-deficient *ob/ob* mice. Leptin, administered either continuously or intermittently (as a daily bolus), increased osteolysis in *ob/ob* mice. Finally, the local bone loss induced by polyethylene particles in *ob/ob* mice treated with leptin recapitulated the histological changes in particle-treated WT mice.

An emerging body of evidence suggests that leptin is required for normal bone resorption in mice. Results reported here and by Bartell *et al*.^30^ demonstrate that leptin administration to *ob/ob* mice increases circulating concentrations of biomarkers for global bone resorption. Recent studies have further demonstrated that *ob/ob* mice exhibit a skeletal phenotype that is consistent with mild osteopetrosis^31^, a disorder characterized by defective bone resorption, resulting in pathological retention of calcified cartilage^32^. Leptin-deficient mice exhibit similar or increased osteoclast surface in cancellous bone compared to WT mice, indicating that osteoclast activity, rather than osteoclast formation, is impaired during leptin deficiency^12,15,33–35^. Thus, impaired osteoclast function in leptin-deficient mice may explain, at least in part, the resistance to polyethylene particle-induced osteolysis in these animals first reported by von Knoch *et al*. in mice housed at room temperature^10^ and confirmed in the present study in mice housed at thermoneutral temperature.

Particle-induced osteolysis differs from resorption associated with normal bone turnover in that the particles act to induce localized inflammation. Inflammation regardless of cause [*e*.*g*., periodontal bone disease^36^, rheumatoid arthritis^37^, gouty arthritis^38^, burns^39^, or particle-induced inflammation^21^] can result in bone loss. Particles act to attract immune cells^40–42^, which in turn, contribute to osteolysis by increasing tissue levels of cytokines that act to increase bone resorption (*e*.*g*., RANKL, TNF-α, IL-1β, IL-17). Inhibitors of RANKL^22^, TNF-α^25,27^, or IL-1β^28,40,43^, or the inhibition of intracellular signaling pathways activated by inflammation (*e*.*g*., NF-κB^25^ or the inflammasome^28^) all act to reduce particle-induced osteolysis in mice. Importantly, many of the cytokines involved in initiation and resolution of inflammation play a role in normal bone turnover.

The leptin receptor is a member of the IL-6 cytokine receptor family^44^ and leptin has been shown to exert actions that affect both innate and adaptive immune responses^44–48^. Leptin acts on macrophages and dendritic cells to increase phagocytosis^17^ and the release of proresorptive cytokines (*e*.*g*., IL-1β, TNF-α, and IL-6)^17,49–51^. Leptin also lowers serum corticosterone levels. This may be important because glucocorticoids in addition to being important in resolution of inflammation are capable of suppressing bone growth and turnover in rodents^52–54^. Therefore, in addition to its actions on normal osteoclastic bone resorption, leptin may also act to increase particle-induced osteolysis through its effects on the immune system. This conclusion is supported by histological changes associated with both particle-induced osteolysis and leptin administration. Specifically, in concordance with von Knoch *et al*.^10^, we provide histological evidence that particles induced less granuloma tissue formation in *ob/ob* mice than in WT mice. In addition, we show that leptin treatment increased granuloma formation. These findings suggest that leptin deficiency reduces and leptin treatment increases the inflammatory response to particles.

The present studies differ from those of von Knoch *et al*.^10^ in that the mice in the current studies were housed at thermoneutral temperature. Sympathetic-driven cold stress induced by standard room temperature housing impairs bone accrual in growing mice and stimulates premature age-related cancellous bone loss. *ob/ob* mice have impaired ability to adapt to cold temperature stress^55^. Therefore, the similarity of response to particle-induced osteolysis in *ob/ob* mice housed at room and thermoneutral temperature argues against higher sympathetic tone in WT mice as being an important factor in particle-induced osteolysis. We have confirmed this conclusion by performing additional temperature studies in which osteolysis did not differ between WT mice housed at 22 °C or 32 °C^56^.

*ob/ob* mice exhibit altered (generally lower) tibial expression of genes related to initiation and regulation of both bone turnover and inflammation. These include cytokines (*e*.*g*., Tnf and Tgfb1, Tgfb2 and Tgfb3), common (Smad4) and receptor-mediated (Smad5) Smads, transcription factors (Gli1, Nfkb1, Phex, Sox9, Twist1), growth factors, (Bmp2, Bmp3, Bmp6, Csf1, Csf3, Pdgfa, Vegfa, Vegfb), growth factor receptors (Bmpr1a), and matrix metalloproteinases (Mmp2, Mmp8)^57^. Thus, similar mechanisms may mediate the reduced bone resorption in *ob/ob* mice associated with normal bone growth and turnover and reduced inflammation-driven bone resorption mediating particle-induced osteolysis. In further support of this concept, physiologically relevant circulating levels of the hormone increased serum CTX in *ob/ob* mice^58^, a finding replicated in the present study in mice treated with the adipokine.

As expected, continuous subcutaneous infusion of leptin exerted effects on energy metabolism in *ob/ob* mice. Leptin-treated *ob/ob* mice lost weight and exhibited lower body fat at sacrifice than particle only *ob/o*b mice. Due to pair-feeding, food consumption did not differ among treatment groups (data not shown). Thus, the weight reduction in mice treated with leptin was most likely due to increased energy expenditure, a well-established response to the hormone^59^. Overall weight reduction in leptin-treated mice over the 2-week period was modest because a large reduction in fat mass was partially compensated for by an increase in lean mass.

In the present studies leptin was administered continuously using subcutaneously implanted osmotic pumps or intermittently by daily subcutaneous injection of the hormone. The former resulted in near normal leptin levels whereas the latter mice would have experienced a sharp rise in leptin shortly after injection followed by a sharp decline due to the adipokine’s short circulating half-life (<1 hr)^60^. Interestingly, both continuous and intermittent leptin treatment exerted similar effects in particle-challenged *ob/ob* mice. Specifically, both treatments acted to increase particle-induced osteolysis, serum CTX and uterine mass, and reduce weight gain, fat mass, blood glucose and serum corticosterone. These findings suggest continuous exposure to leptin is not essential to increase polyethylene-induced osteolysis.

The results of the present studies provide additional evidence that leptin plays a role in mediating polyethylene particle-induced osteolysis. As previously reported^10^, osteolysis was lower in leptin-deficient *ob/ob* mice compared to WT mice. Furthermore, we show that normalizing leptin levels in *ob/ob* mice increases osteolysis to values similar to WT mice. In the future, it will be important to determine whether hyperleptinemia associated with obesity further increases osteolysis or whether lowering leptin levels by dietary restriction or pharmacological treatment reduces the osteolytic response.

In summary, *ob/ob* mice were resistant to polyethylene particle-induced osteolysis when housed at thermoneutral temperature. The impaired osteolytic response was associated with reduced granuloma tissue infiltration, reduced bone resorption, and reduced woven bone formation. Leptin treatment increased the osteolytic response, adding further support to the concept that adipokines contribute to skeletal pathophysiology as well as normal bone physiology. Future research is warranted to determine the precise mechanisms by which leptin regulates normal and pathological bone turnover.

## Materials and Methods

### Experimental protocols

Female *ob/ob* mice on a C57BL/6 (B6) background (stock number 000632) and colony B6 mice (stock number 000664) purchased from Jackson Laboratory (Bar Harbor, Maine) were used in the experiments. Based on genetic background breeding scheme used to maintain the strain, B6 colony animals were recommended by Jackson Laboratory as appropriate controls for *ob/ob* mice. All mice were housed individually in climate-controlled rooms on a 12 hr light dark cycle and fed standard rodent chow (Teklad 8604, Harlen Laboratories, Indianapolis, IN). The animals were maintained in accordance with the National Institutes of Health Guide for the Care and the Use of Laboratory Animals and all animal protocols were approved by the Oregon State University Institutional Animal Care and Use Committee.

### Experiment 1: Effect of leptin deficiency on particle-induced osteolysis in mice housed at thermoneutral temperature

Experiment 1 was conducted to determine whether resistance to polyethylene particle-induced osteolysis observed in *ob/ob* mice housed at room temperature^10^ is also observed in mice housed at thermoneutral temperature. Housing temperature has recently emerged as an important biological variable likely to influence experimental outcomes in mouse studies^61,62^. Mice are conventionally housed at room temperature, which is well below the thermoneutral range for this species. Mice adapt to mild cold stress by adaptive thermogenesis. However, we have shown that increased adaptive thermogenesis during room temperature housing is associated with rapid bone loss in WT mice^20^. *ob/ob* mice have an impaired adaptive response to cold stress^63^. This is reflected by their lower core temperature and decreased expression of UCP-1 in brown fat^64^. Thus, housing temperature may contribute to observed genotype difference in osteolysis in WT and *ob/ob* mice. In Experiment 1, WT B6 and *ob/ob* mice (n = 10/genotype) were housed at thermoneutrality (32 °C) upon arrival at Oregon State University at 4 weeks of age. At 5 weeks of age, the mice within each genotype were randomized by body weight into one of two groups: (1) a no-treatment control or (2) particles, and particles implanted over calvaria. Fluorochromes were administered by subcutaneous injection to label mineralizing bone at time of particle implantation (declomycin, 15 mg/kg; Sigma Chemical, St Louis, MO) and at 4 days and 1 day prior to sacrifice (calcein, 15 mg/kg; Sigma Chemical, St Louis, MO). The mice were sacrificed 2 weeks following particle implantation (at 7 weeks of age). For tissue collection, mice were anesthetized and killed by decapitation. Calvaria were excised, placed in 10% formalin for 24 hours, and transferred to 70% ethanol for storage prior to µCT and histological evaluation.

### Experiment 2: Effect of leptin treatment on particle-induced osteolysis in *ob/ob* mice housed at thermoneutral temperature

Experiment 2 was conducted to determine the effects of leptin treatment on particle-induced osteolysis in *ob/ob* mice. WT (n = 19) and *ob/ob* (n = 30) mice were placed at thermoneutral (32 °C) upon arrival at Oregon State University at 4 weeks of age. From 4 to 6 weeks of age, all *ob/ob* mice were pair-fed to WT mice to control weight gain. At 6 weeks of age, the WT mice were randomized by weight into one of two treatment groups: (1) no-treatment control (n = 10) or (2) particles (n = 9), while the *ob/ob* mice were randomized into one of four treatment groups: (1) no-treatment control (n = 8), (2) particles (n = 8), (3) particles + continuous leptin (cLeptin, n = 6), or (4) particles + intermittent leptin (iLeptin, n = 8), and particles implanted over calvaria. Following particle implantation, all mice were pair fed to the WT + particles group. Mouse leptin (498-OB-05M, R&D Systems, Minneapolis, MN) was delivered continuously (6 µg/d) using subcutaneously implanted osmotic pumps in the *ob/ob* + cLeptin group or intermittently by subcutaneous daily injection (40 µg/d) in the *ob/ob* + iLeptin group. Mice were weighed on day 4, 11, and 14 (necropsy) following particle implantation. Declomycin (15 mg/kg) was administered by subcutaneous injection at 10 days prior to sacrifice and calcein (15 mg/kg) was administered at 3 days and 1 day prior to sacrifice. The mice were sacrificed 2 weeks following particle implantation (at 8 weeks of age). For tissue collection, the mice were anesthetized, weighed, and body composition determined using dual energy absorptiometry (DXA). The animals were then killed by decapitation and trunk blood was collected for evaluation of serum chemistry. Abdominal WAT and uteri were excised and weighed, and calvaria were excised, placed in 10% formalin for 24 hours, and transferred to 70% ethanol for storage prior to µCT and histological evaluation.

### Particle implantation

A polyethylene particle stock solution was prepared to deliver 2.5 mg of particles in 15 µl of solution. Polyethylene particles (S-395 *N1*, Shamrock Technologies Inc., Newark N. J.), mean diameter 5 µm, were washed 6 times in 70% ethanol. Two ml of wet particles were then suspended in 95% ethanol. Particles were placed over the calvarium using a method described by von Knoch^10^. Specifically, mice were anesthetized with 2% isoflurane (Vetone, Meridian, ID) delivered in oxygen using a precision vaporizer (Summit Anesthesia Solutions, Bend, OR). Ophthalmic ointment (Puralube Vet Ointment, Dechra Veterinary Products, Overland Park, KS) was applied to the eyes and Ketofen (5 mg/kg, Fort Dodge Animal Health, Fort Dodge, IA) was injected subcutaneously to alleviate postsurgical discomfort. The surgical area (top of head) was shaved, cleaned with a series of washes (Betadine Scrub, Betadine Solution, and 70% ethanol), and a one cm skin incision was made over the calvarium in the sagittal plane. The skin was retracted and 15 µl of particle solution delivering 2.5 mg of particles in ethanol were applied by pipette on top of the exposed calvarial surface between bregma and lambda. The incision was closed with 7 mm surgical staples (Reflex 7 Wound Closure System). Ketofen (5 mg/kg) was administered once daily for two days following surgery.

### Osmotic pump implantation

Osmotic pumps (Alzet Model 1002, Durect Corporation, Cupertino, CA) were implanted subcutaneously in the particles + cLeptin mice immediately following particle implantation. The dorsal thoracic area was shaved, cleaned, and a one cm incision was made longitudinally across the cleaned region. The osmotic pump was inserted under the skin and the incision was closed with 7 mm surgical staples (Reflex 7 Wound Closure System).

### Densitometry

Body composition (lean mass, fat mass, and percent fat) was assessed by DXA (Piximus2, Lunar Corporation, Madison, WI) under isoflurane anesthesia immediately prior to sacrifice.

### Microcomputed tomography

Calvaria were imaged immersed in 70% ethanol using microcomputed tomography (µCT; µCT40 scanner, Scanco Medical AG, Basserdorf, Switzerland) at 55 kVp x-ray voltage, 145 µA intensity, and 200 ms integration time using 12 µm cubic voxels. Filtering parameters sigma and support were set to 0.8 and 1, respectively. Calvarial osteolysis was evaluated using a semi-quantitative (osteolysis score) assay. Scanned calvaria were imaged at a threshold of ≥235 (scale of 0–1000) and the reconstructed 3-dimensional images used for visual assessment of bone response to particle challenge. Bone response was scored on a scale from 0 (normal bone) to 4 (extensive focal osteolysis) by two blinded independent observers.

### Histology

Following scanning, calvaria were prepared for histological evaluation. The bones were dehydrated in a graded series of ethanol and xylene, and embedded undecalcified in modified methyl methacrylate as described^65^. Coronal sections (4 µm thick) were cut with a vertical bed microtome (Leica 2065) and affixed to gel-coated slides. One section per bone was stained with tartrate resistant acid phosphatase (TRAP) and counterstained with toluidine blue (T-blue) for visualization of cells. A second section was left unstained for visualization of fluorochrome labels.

#### Histopathology score

Histopathology scoring was adapted from a scoring systems we used to evaluate cancer infiltration and resulting bone osteolysis in mice in a model for micro-metastatic tumor growth^66,67^. Tissue response to particles was evaluated separately in (1) unstained sections viewed under ultraviolet light and (2) stained sections viewed under visible light. The analysis was performed on each section using a 0–4 scoring system. In unstained sections, osteolysis and woven bone were evaluated. For osteolysis, a score of 0 represents normal histology whereas scores of 1 to 4 represent progressively increasing levels of pathological periosteal resorption (osteolysis). For woven bone, a score of 0 represents normal histology with no woven bone whereas scores of 1 to 4 represent progressively increasing levels of pathological woven bone formation. Granuloma tissue lining the periosteal surface was evaluated in the stained sections; a score of 0 represents normal histology without granuloma, whereas scores of 1 to 4 represent progressively increasing levels of inflammation.

### Serum chemistry

Blood glucose was measured at necropsy using a glucometer (Life Scan, Inc., Milpitas, CA). Serum leptin was determined using Mouse Leptin Quantikine ELISA Kit (R&D Systems, Minneapolis, MN). Serum CTX was determined using Mouse C terminal telopeptides of type I collagen (CTX1) ELISA kit (Life Sciences Advanced Technologies, St. Petersburg, FL). ELISA were done according to respective manufacturer’s protocol.

### Statistics

Experiment 1 was performed according to a 2 × 2 factorial design with categorical variables for treatment (control and particles) and genotype (WT and *ob/ob*). Two-factor analysis of variance with an interaction between treatment and genotype was used to compare mean values. A linear model with different variances for the different treatment and/or genotype groups was used when variances were distinct. One-factor analysis of variance or the Wilcoxon-Mann-Whitney test was used to analyze data from Experiment 2, with statistical inference focused on comparison of the control *ob/ob*, *ob/ob* + cLeptin, and *ob/ob* + iLeptin groups to the *ob/ob* + particles group. Goodness of fit was evaluated based on Levene’s test for homogeneity of variance, plots of residuals versus fitted values, normal quantile plots, and Anderson-Darling tests of normality. The Benjamini and Hochberg method for maintaining the false discovery rate at 5% was used to adjust for multiple comparisons^68^. Differences were considered significant at p ≤ 0.05. All data are presented as mean ± SE. Data analysis was performed using R version 3.4.3.

## Electronic supplementary material

Figure S1

## Data Availability

All data generated or analyzed during this study are included in this published article.
