# Supplementary material for: Leptin Increases Particle-Induced Osteolysis in Female ob/obMice
Source: Sci Rep. 2018 Oct 4;8:14790. doi: 10.1038/s41598-018-33173-9 (PMC6172200; doi:10.1038/s41598-018-33173-9)
Supplement: Supplementary file 1 — Figure S1 [file 41598_2018_33173_MOESM1_ESM.pdf]

Leptin Increases Particle-Induced Osteolysis in Female *ob/ob* Mice

Kenneth A. Philbrick, PhD[1], Adam J. Branscum PhD[2], Carmen P. Wong PhD[1], Russell T. Turner, PhD[1,3], and Urszula T. Iwaniec, PhD[1,3,]

[1]Skeletal Biology Laboratory, School of Biological and Population Health Sciences, Oregon State University, Corvallis, OR 97331, USA

[2]Biostatistics Program, School of Biological and Population Health Sciences, Oregon State University, Corvallis, OR 97331, USA

[3]Center for Healthy Aging Research, Oregon State University, Corvallis, OR 97331, USA

Supplemental Figure 1. Effects of 2 weeks of leptin administration (continuous, cLeptin; intermittent, iLeptin) on body weight (A), body weight change over the last 10 days of treatment (B), lean mass (C), fat mass (D), percent fat (E), abdominal white adipose tissue (WAT) weight (F), uterine weight (G), blood glucose (H), and serum corticosterone (I) in 8-week-old female *ob/ob* mice. Control and particle-treated WT mice are shown as a reference. Data are mean ± SE; n = 6 - 8/group. [a]Different from *ob/ob* + particles, P < 0.05; [a*]Different from *ob/ob* + particles, P < 0.1.

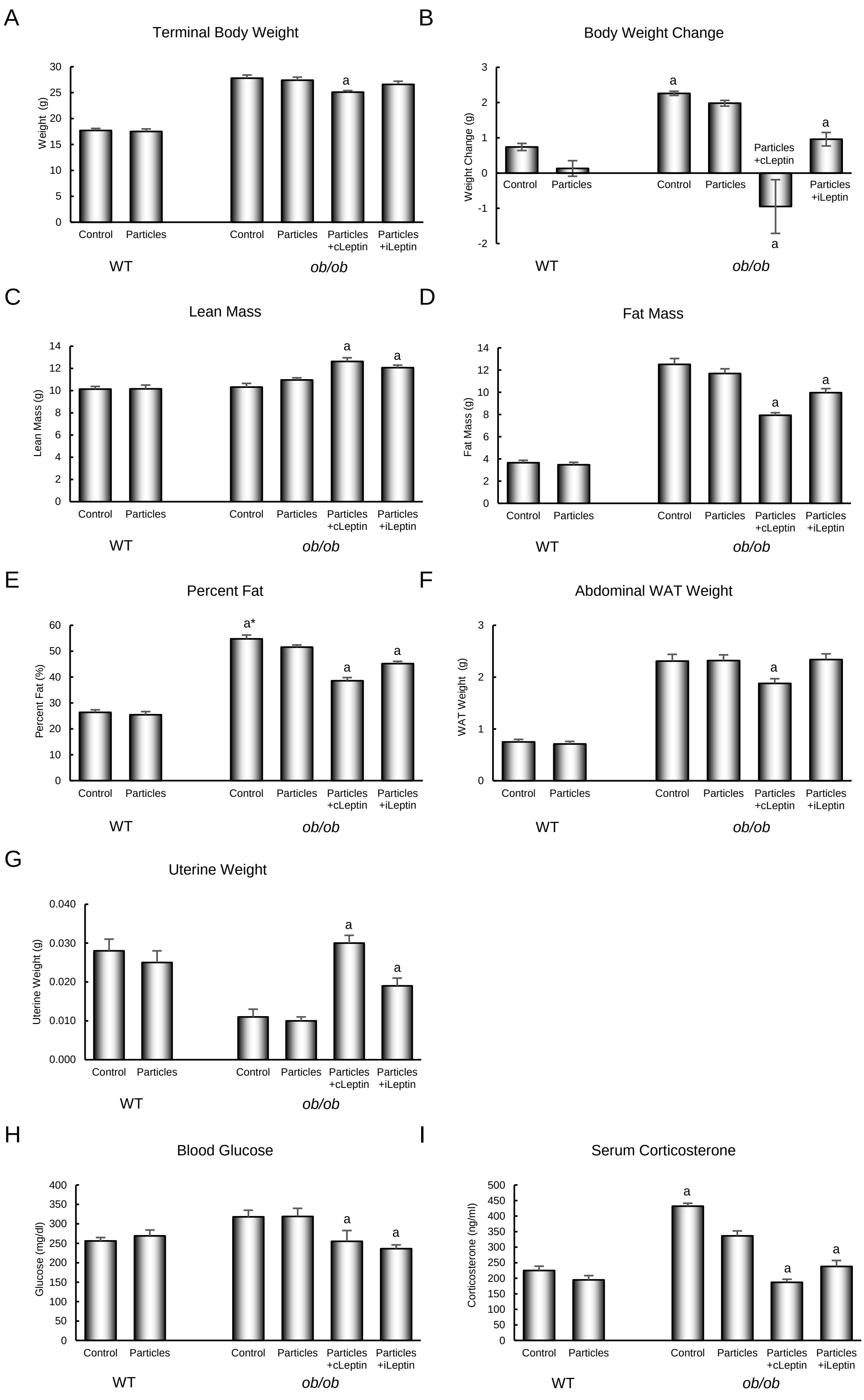
A
Terminal Body Weight
Weight (g)
0
5
10
15
20
25
30
a
Control
Particles
Control
Particles
Particles +cLeptin
Particles +iLeptin
WT
ob/ob
B
Body Weight Change
Weight Change (g)
-2
-1
0
1
2
3
a
a
a
Particles +cLeptin
Particles +iLeptin
Control
Particles
Control
Particles
WT
ob/ob
C
Lean Mass
Lean Mass (g)
0
2
4
6
8
10
12
14
a
a
Control
Particles
Control
Particles
Particles +cLeptin
Particles +iLeptin
WT
ob/ob
D
Fat Mass
Fat Mass (g)
0
2
4
6
8
10
12
14
a
a
Control
Particles
Control
Particles
Particles +cLeptin
Particles +iLeptin
WT
ob/ob
E
Percent Fat
Percent Fat (%)
0
10
20
30
40
50
60
a*
a
a
Control
Particles
Control
Particles
Particles +cLeptin
Particles +iLeptin
WT
ob/ob
F
Abdominal WAT Weight
WAT Weight (g)
0
1
2
3
a
Control
Particles
Control
Particles
Particles +cLeptin
Particles +iLeptin
WT
ob/ob
G
Uterine Weight
Uterine Weight (g)
0.000
0.010
0.020
0.030
0.040
a
a
Control
Particles
Control
Particles
Particles +cLeptin
Particles +iLeptin
WT
ob/ob
H
Blood Glucose
Glucose (mg/dl)
0
50
100
150
200
250
300
350
400
a
a
Control
Particles
Control
Particles
Particles +cLeptin
Particles +iLeptin
WT
ob/ob
I
Serum Corticosterone
Corticosterone (ng/ml)
0
50
100
150
200
250
300
350
400
450
500
a
a
a
Control
Particles
Control
Particles
Particles +cLeptin
Particles +iLeptin
WT
ob/ob
